# Do good actions inspire good actions in others?

**DOI:** 10.1038/srep07470

**Published:** 2014-12-12

**Authors:** Valerio Capraro, Alessandra Marcelletti

**Affiliations:** 1 Center for Mathematics and Computer Science (CWI), 1098 XG, Amsterdam, The Netherlands; 2 Department of Economics, University of Rome ‘Tor Vergata’, 00133, Roma, Italy

## Abstract

Actions such as sharing food and cooperating to reach a common goal have played a fundamental role in the evolution of human societies. Despite the importance of such *good* actions, little is known about if and how they can spread from person to person to person. For instance, does being recipient of an altruistic act increase your probability of being cooperative with a third party? We have conducted an experiment on Amazon Mechanical Turk to test this mechanism using economic games. We have measured willingness to be cooperative through a standard Prisoner's dilemma and willingness to act altruistically using a binary Dictator game. In the baseline treatments, the endowments needed to play were given by the experimenters, as usual; in the control treatments, they came from a good action made by someone else. Across four different comparisons and a total of 572 subjects, we have never found a significant increase of cooperation or altruism when the endowment came from a good action. We conclude that good actions do not necessarily inspire good actions in others. While this is consistent with the theoretical prediction, it challenges the majority of other experimental studies.

Humans are unique in the animal world for their willingness to help others and collaborate to reach a common goal and these attitudes are among the main reasons why human societies are so successful^1^,^2^,^3^,^4^,^5^,^6^,^7^,^8^,^9^. Good actions, those which maximise the other's payoff, abound in the everyday life and experiments with anonymous people show that they are common even in the ideal setting of a lab, where confounding factors such as long-term strategies, indirect rewards, communication, signalling, etc., are not present^10^,^11^,^12^,^13^,^14^,^15^,^16^.

Nevertheless, not everyone behaves in such good ways and clouds of selfish people can often be observed in both the everyday life and the lab^17^,^18^. Some people perceive the individual cost of restricting their individual freedom of choice as being too large and decide to free-ride and aim for their personal benefit; consequently, people in connections with these free-riders typically decide to either break the link (via active linking^19^, when allowed) with the free-riders or to free-ride as well (via direct reciprocity^7^). Either way, this generates a cloud of defectors in the human social network.

To avoid this suboptimal scenario, scholars have started investigating what mechanisms can promote the evolution of good actions in lab experiments. The underlying motivation is that, in case we knew that mechanism M promotes such behaviours, then institutions could use this mechanism to foster good actions and give rise to more successful societies.

Here we consider three different kinds of good actions: altruism, benevolence, and cooperation. *Altruism* is defined as paying a cost to increase the benefit of someone else. We measure altruistic attitudes through the (binary) Dictator game (DG)^20^. Here, one person, named dictator, is given an endowment of 100 Monetary Units (MUs), while the other person is given nothing. The dictator can either take all the 100 MUs for himself and leave the other person with nothing or he can split the money evenly. The other person has no choice and receives what the dictator decides to donate. *Benevolence* is defined as a strong form of altruism, where you make an action that not only increase the other person's payoff, but also the final payoff of the other person is larger than yours^15^. We measure benevolent characteristics through the Benevolence game (BG)^15^. Here a person is given an endowment of 100 MUs and has to decide between burning it or give it to another player. Also in this case, the other person has no say. Finally, *cooperation* is defined as reciprocal altruism, where you pay a cost to increase someone else's benefit, but the other person has the opportunity to reciprocate your good action. We measure cooperative tendencies through the standard Prisoner's dilemma (PD). Here two people are given 100 MUs each and have to decide between hand it over or not. If a participant hands his money over, the other participant earns 100 *k* MUs, where *k* > 1. In the DG, it is optimal to keep all the money, but the good action is to split it; in the BG a money maximiser person is indifferent between his two strategies, while the good action is to let the other person get the endowment; in the PD, participants are better off not handing the money over, but the good action is to hand it over. Altruism, benevolence, and cooperation are often correlated^14^,^15^, but they are not the same. For instance, in the BG we usually see about 80% of subjects acting benevolently, while in the DG and the PD the percentage of people acting in a good way is often below 50%.

Many studies have been focused on whether mechanisms such as punishment of defectors^21^,^22^,^23^,^24^, reward of cooperators^25^,^26^,^27^ or a combination of these two^28^,^29^,^30^,^31^ can promote cooperative behaviour. Little is known about if and how good actions of possibly different nature can spread from person to person to person. For instance, does being recipient of an altruistic action increase your probability to cooperate with a third party? Answering this kind of questions via lab experiments with anonymous participants is particularly relevant now that we live in a society where a ‘substantial part of our life is spent in the company of strangers and transactions are no longer face-to-face’^32^.

Plato's quote ‘Good actions give strength to ourselves and inspire good actions in others’ suggests that recipients of good actions should act in a better way with others than people who were recipient of a neutral action. Besides the clear applications that this principle, if true, would have on finding ways to foster good behaviours in human societies, it may potentially have deep consequences also in economic theory: if our actions strongly depend on what others have previously done to us, the stability of our preferences over time would be seriously questioned. Perhaps we were selfish yesterday because someone was mean to us in the early morning, and we are altruist today because someone has been kind to us this morning.

Making this problem even more intriguing is the current discrepancy between theoretical predictions and experimental data. From a theoretical point of view, upstream reciprocity^33^,^34^, the particular kind of indirect reciprocity^32^,^35^ that allows the spread of a good action from person to person to person, has been shown *not* to lead to the evolution of cooperation, predicting that Plato's principle has no hope to find empirical support. Yet, with one exception^36^, finding that Person B is *not* more likely to cooperate in the Public Goods game after being recipient of a cooperative action from one or more Person(s) A, the few experimental studies that have been performed so far generally agree on the fact that good actions actually spread from Person A to Person B to Person C^37^,^38^,^39^,^40^,^41^,^42^,^43^,^44^,^45^. One of these studies^46^ suggests that the spread of cooperation may depend on the underlying network structures: cooperative behaviour spreads from Person A to Person B to Person C in fixed networks, but not in dynamic networks.

Here we add to this experimental literature and go beyond it in the following way. Our typical experiment involves three people, A, B, and C. The description of the exact design is postponed to the Methods section. Abstractly speaking, all our experiments have the same basic structure: Person B is, at the same time, the target of a decision made by Person A, and the maker of a decision that can affect only Person C. We investigate whether the nature of A's action (good or neutral) affect the nature of B's action. Since we are interested in seeing how B's altruistic and cooperative tendencies change given the choice of A, Person B will either play a Dictator game or a Prisoner's dilemma. This gives rise to two baseline treatments, where B is asked to play either the DG or the PD, after being the recipient of a neutral action (endowment needed to play given by the experimenter). While previous studies have focused on the spread of the *same* good action, here, motivated by more realistic scenarios, we also study the spread of good actions of possibly *different* nature. Specifically, by varying the way the endowment is given to Person B by Person A (either an altruistic act in the DG or a benevolent act in the BG) we created four control treatments, where B is asked to play either the DG or the PD after being the recipient of a good action. Thus, besides addressing the question of whether altruism in the dictator game spreads, which was already done in refs. 38 and 41, we can also address three novel research questions: Does being recipient of an altruistic action increase your probability to cooperate with a third party? Does being recipient of a benevolent action increase your probability to cooperate with a third party? Does being recipient of a benevolent action increase your probability to act altruistically in favour of a third party?

Detailed statistical analysis will be reported in the Results section, but we anticipate that, in all four comparisons and a total of 572 subjects, we have never found statistically significant difference in the behaviour of B towards C, depending on how A behaved towards B. Thus our results are in line with the theoretical predictions, but they challenge the experimental results reported in refs. 38 and 41. In the Discussion section we will compare our study with these two and describe what reasons could have driven this discrepancy.

## Methods

We performed two studies. In Study 1 we have investigated whether recipients of a good action behave more cooperatively than recipients of a neutral action. In the baseline treatment, subjects in the role of Person B participated in a neutrally framed one-shot PD. Each participant was given an endowment of $0.20 and paired with another anonymous participant (Person C). They could either keep the $0.20 or hand it over. In this latter case, the other participant would earn $0.40. The two control treatments differed from the baseline treatment only in the way the initial $0.20 was given to the participants. In Control 1, participants were informed that they had been previously paired with another participant (Person A), different from Person C, who was the dictator in a DG. Person A was given $0.40 and could decide between keeping it all or splitting it with Person B and they decided to split it. Thus, in Control 1, the endowment needed to play the PD comes from an altruistic act of Person A. Control 2 was similar to Control 1, with the only difference that the $0.20 comes from a BG. Specifically, participants were informed that they had been previously paired with another anonymous participants (Person A) who had to decide between doing nothing or making the benevolent act of letting the other player getting $0.20 at zero cost to themselves. After making their decision, in each treatment participants entered the demographic questionnaire, where we asked for their gender, age, level of education and, finally, the reason of their choice in the game.

In Study 2 we have tested whether recipients of a good action behave more altruistically than recipients of a neutral action. In the baseline treatment, subjects participated to a binary DG. Each participant was given an endowment of $0.20 and had to decide between keeping it and splitting it evenly with the other participant. Also in this case, the two control treatments differed from the baseline only in the way the initial $0.20 was provided. In Control 1 they came from a donation in a previous DG; in Control 2 they came from a benevolent act in a previous BG. As in Study 1, after making their decision, subjects enter the demographic questionnaire, where we asked for their gender, age, level of education, and the reason of their choice in the game.

We recruited US residents using the online labour market Amazon Mechanical Turk (AMT)^47^,^48^,^49^. As in classical lab experiments, AMT workers receive a baseline payment ($0.30 in our case) and can earn an additional bonus depending on how they perform in the game. AMT experiments are easy to implement and cheap to realise, since AMT workers are paid a substantially smaller amount of money than people participating in physical lab experiments. Nevertheless, it has been shown that data gathered using AMT agree both qualitatively and quantitatively with those collected in physical labs in a variety of different strategical situations^17^,^36^,^48^,^49^.

Yet there are some issues that may potentially invalidate data obtained using AMT. One of the major issues is that some subjects try to complete the game as fast as they can, to get the bonus in the shortest time, and so they may not fully understand the strategic situation that they are facing, increasing the risk of collecting meaningless data. We have addressed this problem in two different ways, depending on whether subjects acting as Player B had to play the DG or the PD. In the PD, we asked for four comprehension questions and we automatically screened out those subjects who failed any of the comprehension questions. Comprehension questions were formulated in order to make clear the tension between maximising one's own payoff and maximising the other's payoff. In the DG, since the strategical situation is really straightforward, we decided to skip the comprehension questions and ask the subjects to describe the reason of their choice. This procedure allowed us to check whether the subjects understood the decision problem, but also to address the second major issue of AMT experiments, namely that some subjects may think that the participants they are paired with are not real. Indeed, typically, in the description of the reason of their choice, one finds a few subjects saying ‘I kept the money because I think the other person is not real’, or similar statements. Of course, we would like to exclude systematically these problematic subjects. To do so, we have manually checked all the original DataSet and we have constructed DataSetExcluded by removing those subjects belonging to one of the following categories: (i) Subjects who explicitly say that they believe that the other participant is not real; (ii) Subjects who do not provide any reason for their choice; (iii) Subjects whose reason of their choice was not consistent with their actual choice. This led us with two datasets to analyse: DataSet and DataSetExcluded. Statistics are reported in the Results section, but we anticipate that the two analyses turned out to be qualitatively equivalent.

After collecting the results, subjects were matched and bonuses were computed and paid. No deception was used. Informed consent was obtained by all participants. These experiments were approved by the Southampton University Ethics Committee on the Use of Human Subjects in Research and carried out in accordance with the approved guidelines.

## Results

**Figure 1:**
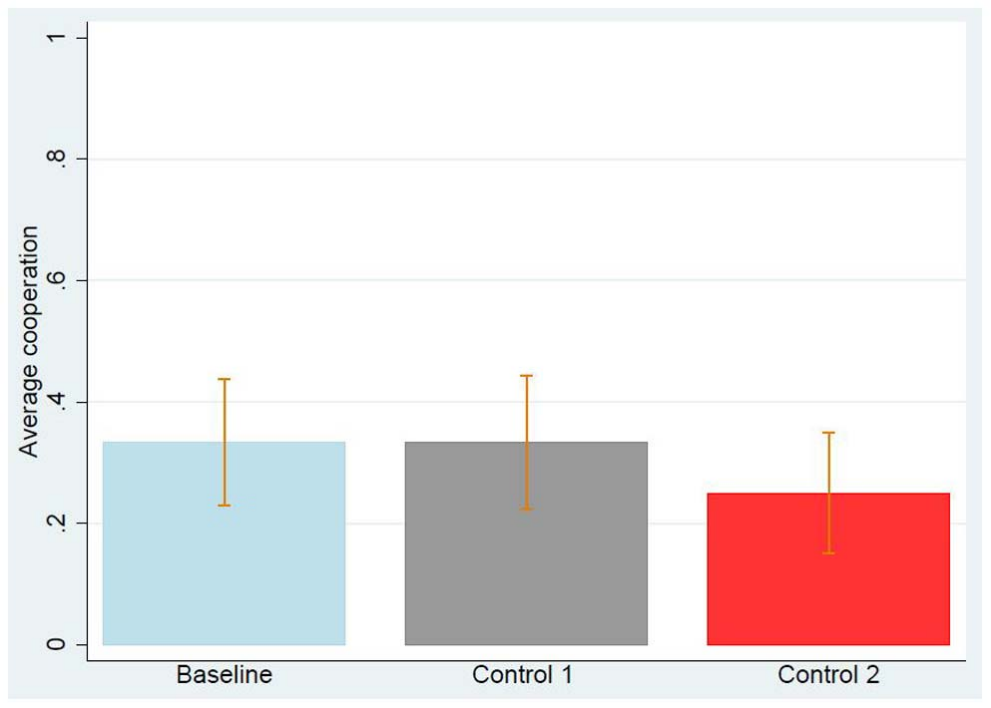
Average cooperation in Study 1 for each of the three treatments, using DataSet. Error bars represent the standard error of the mean. It is visually clear that the control treatments do not significantly differ from the baseline. Logistic regression confirms this expectation, as reported in Table 1.

A total of 232 US subjects (62.9% male, mean age = 30.4) participated in our Study 1, as Person B, passing all comprehension questions. 81 subjects (64.2% male, mean age = 31.1) participated in the baseline and played a neutrally framed PD using an endowment provided by the experimenter; 75 subjects (58.7% male, mean age = 30.4) participated in Control 1 and played the PD using an endowment coming from a donation in a previous DG; 76 subjects (65.8% male, mean age = 29.7) participated in Control 2 and played the PD using an endowment coming from a benevolent act in a previous BG. The average cooperation is very similar across the three treatments (33.3% in the baseline, 33.3% in Control 1, 25.0% in Control 2). See Figure 1. To test for an effect of how the endowment is provided (baseline vs Control 1 and baseline vs Control 2) we use logistic regression, with and without control on gender, age, and level of education, predicting cooperation or defection as the dependent variable. As shown by Table 1 we find no significant effect of how the endowment was provided. Table 2 shows the effect of demographics on cooperation: none of the demographic characteristics we collected predicts cooperation significantly. As mentioned in the Methods section, we now report the statistical analysis of DataSetExcluded. In a manual screening, we excluded 9 subjects and remained with 223 US subjects (62.8% male, mean age = 30.5). 79 subjects (62.9% male, mean age = 31.3) in the baseline, 71 subjects (60.6% male, mean age = 30.4) in Control 1, and 74 subjects (64.9% male, mean age = 29.8) in Control 2. Also in this case, the average cooperation was very similar across the three treatments (33.3% in the baseline, 32.3% in Control 1, and 25.7% in Control 2) and Table 1 shows that the way the endowment was provided had no statistically significant effect on cooperative behaviour. We conclude, that a good action in the DG or in the BG does not inspire a good action in the PD, at least in our subject pool. This conclusion is also supported by the fact that none of the subjects in the control treatments declared that his or her action in the PD was somehow influenced by how they were previously treated.

**Figure 2:**
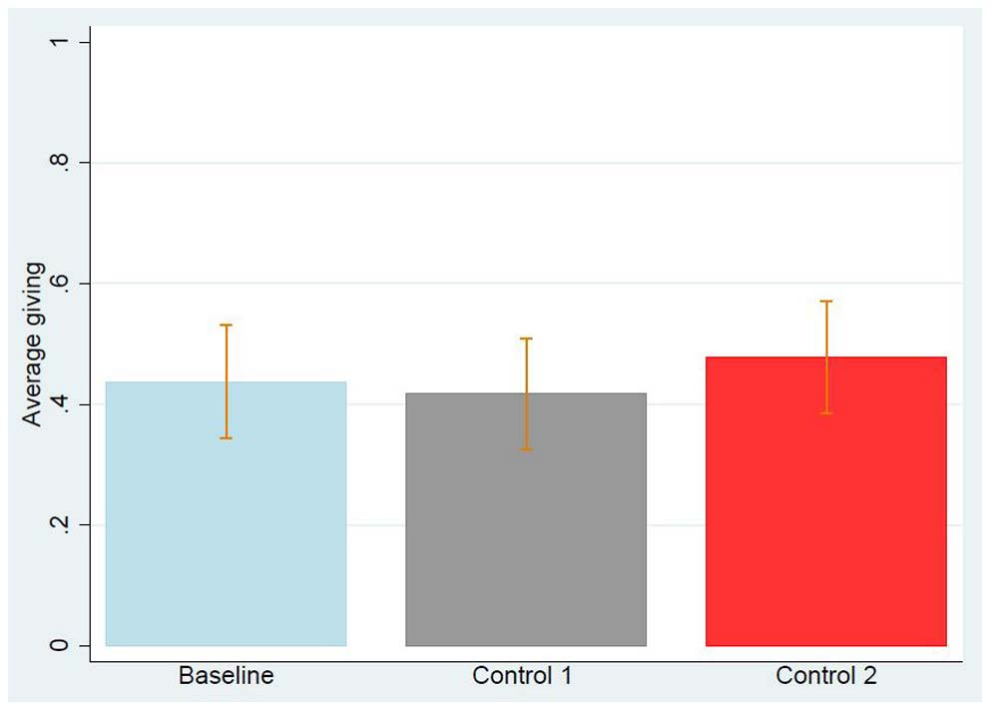
Average giving in Study 2 for each of the three treatments, using DataSet. Error bars represent the standard error of the mean. It is visually clear that the control treatments do not significantly differ from the baseline. Logistic regression confirms this expectation, as reported in Table 3.

A total of 340 US subjects (63.9% male, mean age = 29.2) participated in our Study 2, as Person B. 112 subjects (59.8% male, mean age = 28.1) participated in the baseline and played a neutrally framed binary DG with endowment provided by the experimenter; 115 subjects (57.4% male, mean age = 28.7) participated in Control 1 and played the binary DG with endowment coming from a donation in a previous binary DG; 113 subjects (74.4% male, mean age = 30.7) participated in Control 2 and played the binary DG with endowment coming from a benevolent act in a previous BG. The average choice (Keep = 0, Split = 1) is very similar across the three treatments (0.43 in the baseline, 0.42 in Control 1, 0.48 in Control 2). See Figure 2. To test for an effect of how the endowment is provided (baseline vs Control 1 and baseline vs Control 2) we use logistic regression, with and without control on gender, age, and level of education, predicting keeping or splitting as the dependent variable. As shown by Table 3 we find no significant effect of how the endowment was provided. Table 2 shows the effect of demographics on cooperation: females donated significantly more than males and age had a borderline significant positive effect on altruism. As mentioned in the Methods section, we now report the statistical analysis of DataSetExcluded. In a manual screening, we excluded 24 subjects and remained with 316 US subjects (62.7% male, mean age = 29.3). 108 subjects (58.3% male, mean age = 28.1) in the baseline, 102 subjects (54.9% male, mean age = 29.3) in Control 1, and 106 subjects (74.5% male, mean age = 30.5) in Control 2. Also in this case, the average choice was very similar across the three treatments (0.45 in the baseline, 0.47 in Control 1, and 0.50 in Control 2) and Table 1 shows that the way the endowment was provided had no statistically significant effect. We conclude, that a good action in the DG or in the BG does not inspire a good action in the DG, at least in our subject pool. We mention that, this time, several participants declared that they were influenced by how they were previously treated. To be more precise, 15 out of 102 subjects in Control 1 Excluded declared ‘The other participant chose to split his 40 cents with me, so I elected to pass on the love and do the same’ or equivalent statements. A similar thing happened in Control 2. Thus there is a psychological effect, but it does not give rise to an economically and statistically significant effect. It is then possible that those people who reported to be positively influenced by the other's choice would split the money anyway.

Finally, a total of 175 US subjects were recruited to play the role of Person A (90 in the DG and 85 in the BG). Note that the statistics in Table 2 do not include these participants, since they played a different DG ($0.40 at stake, instead of $0.20). Statistics on these subjects are not significant, probably due to the relatively small sample. We used these subjects only to avoid deception and match the players in the role of Person B with a real participant.

## Discussion

Good actions are defined as those which maximise the other's payoff. Sharing the endowment in the binary Dictator game (DG), letting the other person take the endowment in the Benevolence game (BG), cooperating in the Prisoner's dilemma (PD), are all examples of good actions. Motivated by Plato's quote ‘Good actions give strength to ourselves and inspire good actions in others’ we have investigated whether good actions of possible different types can spread from person to person to person in the simplest possible way: does a good action of A towards B increase the probability of a good action of B towards C? We have conducted six experiments: two baseline treatments where B has to make a decision in either the DG or the PD using an endowment given by the experimenter; four control treatments where the endowment needed to play comes from either an altruistic action or a benevolent action made by someone else in a previous interaction. Across four comparisons and 572 participants we have never found a significant increase of good actions by player B when they were recipient of a good action.

Our results provide evidence that good actions do not spread from person to person to person and thus they support the theoretical prediction that upstream reciprocity alone does not lead to the evolution of good actions^34^. However, they challenge the experimental results reported in refs. 38 and 41, which also analyse the spread of altruistic behaviour in the DG.

In ref. 38, the authors show that altruistic behavior spreads when people interact fact-to-face. There is reason to believe that face-to-face interactions are profoundly different from anonymous interactions and this may have driven the spread observed in ref. 38. Indeed, face-to-face good actions are typically accompanied by eye-contact, small talks, smiles, and other kinds of signals, which are likely to improve the recipient's mood so that the recipient's utility is the sum of his material payoff and his non-material payoff given by the fact that his mood has been indirectly improved by other factors. It is then possible that it is not really the fact of being recipient of a good action that inspires other good actions, but the fact that the mood has been improved by other factors.

Closer to our experiment is the one reported in ref. 41, where the authors find evidence of the spread of altruistic actions in an anonymous setting. Here we can only conjecture a reason of the discrepancy between our results and theirs. In the setting of ref. 41, recipients of a good action had a larger endowment to spend and so it is a priori possible that larger givings from recipients of good actions were driven by having just more money to spend. The authors tried to exclude this possibility in their Experiment 2, but they compared only those who were recipient of a bad action with those who were recipient of a good action and it is then not clear where the recipients of a neutral action are seated. Moreover, when they analysed whether the endowment had driven their effect, they found a non-significant but rather small p-value (0.12), when compared with the sample size, that was rather small as well (100 participants, divided in four treatments). It is then possible that the same experiment, with a little larger sample, would have given negative results; or, alternatively, that comparing also the recipients of a neutral action would have shown that their effect was driven by a strong decrease on the givings from those who were recipient of a bad action, rather than an increase from those who were recipient of a good action.

Further investigation are then needed to solve this discrepancy, also in light of the fact that our experiment as well has its own limitations, the major of which is that it is virtually impossible to convince AMT workers that the people they interact with are real. To address this problem, we asked the subjects to write the reason of their choice and conducted two statistical analyses, one including all participants and one including only those whose description revealed that they were playing as they would do in reality. Although the two analyses gave qualitatively equivalent results, we cannot be completely sure that *all* participants in the second analysis acted as they would act in a real scenario: it is possible that they described a reason consistent with a real scenario, but behaved as the other participants were not real. On the other hand, it is extremely difficult to design an experiment where participants are made completely convinced that the other participants are real, without generating confounding factors. For instance, showing pictures, names, or TurkIDs would decrease the ‘distance’ between the participants with the consequence of increasing good actions, as shown by a number of similar studies^50^,^51^,^52^.

In addition to what described above, our results also contribute to the increasing body of literature regarding gender differences in the Dictator game. Alike the majority of studies^53^,^54^,^55^,^56^,^57^,^58^,^59^,^60^, but not all^57^,^60^,^61^, we too have found that females are significantly more generous than males in the Dictator game. Furthermore, consistent with Engel's metastudy^62^, we too have found that age has a positive effect on giving in the DG, though our effect is only borderline significant (*P* = 0.05 in DG and *P* = 0.092 in DG excluded).

Finally, our results add to the research concerning framing effects in the Dictator game and the Prisoner's dilemma. A number of studies^57^,^59^,^63^ agree that behaviour in the Dictator game is independent of the name of the game (Keep game vs Take game) and the name of the strategies (Keep vs Give). Here we have shown that it is independent of how the endowment is provided (by the experimenter vs by someone else through a good act). Similarly, a number of studies^64^,^65^,^66^,^67^,^68^,^69^,^70^,^71^,^72^,^73^,^74^,^75^, but not all^76^,^77^,^78^, suggest that behaviour in the Prisoner's dilemma depends on the name of the game and the name of the strategies. Here we have provided evidence that it does not depend on how the endowment is provided.

## Author Contributions

V.C. designed and performed the experiment, V.C. and A.M. analysed the data and wrote the manuscript.

## Supplementary Material

Supplementary Information Supplementary Information

## Figures and Tables

**Table 1:** The effect of being recipient of a good action on cooperation in the Prisoner's Dilemma. We used logistic regression with and without control on sex, age, and education, using ‘treat’ as a dummy variable. We report the *β*-value, its standard error, and the significance level. ‘Bas’ stands for ‘Baseline’, ‘Con’ for ‘Control’, and ‘Exc’ for ‘Excluded’. So, for instance, the column ‘Bas Exc vs Con 1 Exc’ reports the results of the regression using Bas Exc = 0 and Con 1 Exc = 1 as dummy variable. We find no significant effect of the dummy variable on cooperation in the PD. Being recipient of a good action does not significantly increase the probability of cooperating in the PD

	Bas vs Con 1	Bas vs Con 1	Bas Exc vs Con 1 Exc	Bas Exc vs Con 1 Exc	Bas vs Con 2	Bas vs Con 2	Bas Exc vs Con 2 Exc	Bas Exc vs Con 2 Exc
dummy for decision								
treat	−.000	.043	−.043	.022	−.405	−.419	−.370	−.382
	(.34)	(.34)	(.35)	(.36)	(.36)	(.36)	(.36)	(.37)
sex		.020		−.065		−.198		−.227
		(.35)		(.37)		(.38)		(.39)
age		−.012		−.017		−.004		−.004
		(.02)		(.02)		(.02)		(.02)
education		.291		.394*		.252		.308
		(.16)		(.16)		(.17)		(.18)
Constant	−.693**	−1.620	−.693**	−1.800	−.693**	−1.399	−.693**	−1.595
	(.24)	(.99)	(.24)	(1.04)	(.24)	(1.02)	(.24)	(1.03)
Pseudo *R*^2^	.000	.019	.000	.034	.007	.019	.006	.024
No. of cases	156	156	149	149	157	157	152	152

**Significance level**:

**: *p* < 0.01.

*: *p* < 0.05.

**Table 2:** Impact of demographic variables on individual decision in the DG and the PD before and after exclusion of subjects. We used logistic regression and report the *β*-value, its standard error, and its significance level. Females donate significantly more than males in the DG. Borderline positive effects of age on altruism in the DG and education on cooperation in the PD were also noted

	DG	DG Exc	PD	PD Exc
dec				
sex	.671***	.594**	.136	.060
	(.18)	(.19)	(.30)	(.30)
age	.015*	.017	−.001	−.004
	(.01)	(.01)	(.01)	(.01)
education	−.092	−.150	.209	.278*
	(.08)	(.08)	(.14)	(.14)
Constant	−1.140*	−.711	−1.886*	−1.994*
	(.46)	(.48)	(.81)	(.84)
Pseudo *R*^2^	.029	.029	.010	.015
No. of cases	340	316	232	223

Significance level:

***: *p* < 0.001.

**: *p* < 0.01.

*: *p* < 0.05.

**Table 3:** The effect of being recipient of a good action on altruism in the Dictator game. We used logistic regression with and without control on sex, age, and education, using ‘treat’ as a dummy variable. We report the *β*-value, its standard error, and the significance level. ‘Bas’ stands for ‘Baseline’, ‘Con’ for ‘Control’, and ‘Exc’ for ‘Excluded’. So, for instance, the column ‘Bas Exc vs Con 1 Exc’ reports the results of the regression using Bas Exc = 0 and Con 1 Exc = 1 as dummy variable. We find no significant effect of the dummy on donations in the DG. Being recipient of a good action does not significantly increase the probability of splitting the endowment in the DG

	Bas vs Con 1	Bas vs Con 1	Bas Exc vs Con 1 Exc	Bas Exc vs Con 1 Exc	Bas vs Con 2	Bas vs Con 2	Bas Exc vs Con 2 Exc	Bas Exc vs Con 2 Exc
dec								
treat	−.082	−.115	.068	.024	.163	.257	.186	.289
	(.27)	(.27)	(.28)	(.28)	(.27)	(.28)	(.27)	(.29)
sex		.545		.436		.624*		.619*
		(.28)		(.29)		(.31)		(.31)
age		.030		.028		.015		.015
		(.02)		(.02)		(.02)		(.02)
education		.047		−.006		−.194		−.229
		(.11)		(.12)		(.11)		(.12)
Constant	−.251	−2.078**	−.186	−1.556*	−.251	−.746	−.186	−.520
	(.19)	(.74)	(.19)	(.76)	(.19)	(.74)	(.19)	(.77)
Pseudo *R*^2^	.000	.032	.000	.023	.001	.032	.002	.035
No. of cases	227	227	210	210	225	225	214	214

**Significance level**:

**: *p* < 0.01.

*: *p* < 0.05.
