# Supplementary material for: Do good actions inspire good actions in others?
Source: Sci Rep. 2014 Dec 12;4:7470. doi: 10.1038/srep07470 (PMC4264010; doi:10.1038/srep07470)
Supplement: Supplementary Information [file srep07470-s1.pdf]

SUPPLEMENTARY INFORMATION FOR THE PAPER 'DO GOOD ACTIONS INSPIRE GOOD ACTIONS IN OTHERS?'

VALERIO CAPRARO[1] & ALESSANDRA MARCELLETTI[2]

[1]Center for Mathematics and Computer Science (CWI), 1098 XG, Amsterdam, The Netherlands. V.Capraro@cwi.nl.[2]Department of Economics, University of Rome 'Tor Vergata', 00133, Roma, Italy.

Full Instructions of Study 1

Here we report the full instructions for Control 1. The instructions for Baseline and Control 2 were very similar and we highlight the differences along the description.

The first two screens do not contain any information about the game and serve us only as control to avoid multiple plays from the same subject and lazy participants who can increase randomness on our data.

Screen 1. In the first screen, participants were welcomed to the game and asked to type their worker ID. This allows us to automatically exclude workers who have already completed the task.

Screen 2. In the second screen, we asked the participant to transcribe a relatively long neutral piece of text. This allows us as to tell computers and humans apart (CAPTCHA) and, at the same time, to exclude lazy workers and minimise randomness in our data. We used a meaningless neutral text in order to avoid framing effects.

In the third screen, people entered the real game. Here is the exact instructions we used.

Screen 3. Welcome to this HIT. This HIT will take about ten minutes. For the participation to this HIT, you will earn $0.30. You can also earn additional money depending on the decision you and the other participants will make. You will be asked to make two decisions. There is no incorrect answer. However, to make sure you understand the situation, we will ask some simple questions, each of which has only one correct answer. If you fail to correctly answer any of those questions, the survey will automatically end and you will not receive any redemption code and consequently you will not get any payment. With this in mind, do you wish to continue?

Here participants could either continue or end the survey, clicking on the corresponding button. Participants who decided to continue were directed to the next screen.

Screen 4 (Control 1) In a previous part of the HIT, which you have not seen, you were paired with another participant. This participant was given $0.40 and had to decide between keeping it all or splitting it evenly with you. He decided to split. So.. congratulations! You now have $0.20.

Screen 4 (Control 2) In a previous part of the HIT, which you have not seen, you were paired with another participant. The other participant was told that there were $0.20 available but she or he could not get it. They could only choose between doing nothing or donating it to you. He decided to donate. So.. congratulations! You now have $0.20.

Screen 4 (Baseline) In the Baseline treatment participants jumped from Screen 3 to Screen 5.

Screen 5. You have been paired with another participant, different from the one you were paired before. The amount of money you can earn depends on your and the other participant's decision. You and the other participant have both $0.20, earned in the previous part of the HIT. You must decide whether to hand it over or not. Each time a participant hands over their $0.20, the other participant earns $0.40. So:

(1) If you both decide to hand over the $0.20, you end the game with $0.40
(2) If the other participant hands it over and you do not, you end the game with $0.60
(3) If you hand it over and the other participant does not, you end the game with $0
(4) If neither of you hand it over, then you end the game with $0.20

Of course, in the Baseline, we took out the sentence 'different from the one you were paired before'.

Screen 6. Here are some questions to ascertain that you understand the rules. Remember that you have to answer all of these questions correctly in order to get the completion code. If you fail any of them, the survey will automatically end and you will not get any payment.

(1) What choice should YOU make to maximise YOUR gain?
(2) What choice should YOU make to maximise the OTHER PARTICIPANT's gain?
(3) What choice should the OTHER PARTICIPANT make to maximise THEIR gain?
(4) What choice should the OTHER PARTICIPANT make to maximise YOUR gain?

In each questions, participants could answer by selecting either 'Do not hand over' or 'Hand over'. Participants failing any of the comprehension questions were automatically screened out through a 'Skip Logic', which is very easy to implement using the survey builder Qualtrics.

Screen 7. Now it's time to make your decision. What is your choice?

Here participants could select either 'Do not hand over' or 'Hand over'. Following this screen, we asked demographic questions and the description of the reason of their choice. A final screen, providing a completion code to claim for their payment, concluded the survey.

Full Instructions of Study 2

Here we report the full instructions for Control 1. The instructions for Baseline and Control 2 were very similar and we highlight the differences along the description. Moreover, since some screens were identical to those of Control 1 in Study 1, we report only those screen containing some differences.

Screen 5. You have been paired with another participant, different from the one you were paired before. You must decide between keeping all your $0.20 or splitting it evenly with the other participant. This decision is unilateral. The other participant does not have the possibility to influence your payoff.

Of course, in the Baseline, we took out the sentence 'different from the one you were paired before'.

Screen 6. As mentioned in the Main Text, Study 2 does not contain any comprehension questions. So subjects passed directly from Screen 5 to Screen 7.

Screen 7. What is your choice?

Here participants could select either 'Keep' or 'Split'. Following this screen, we asked demographic questions and the description of the reason of their choice. A final screen, providing a completion code to claim for their payment, concluded the survey.
